# Comprehensive characterization of the DNA amplification at 13q34 in human breast cancer reveals TFDP1 and CUL4A as likely candidate target genes

**DOI:** 10.1186/bcr2456

**Published:** 2009-12-08

**Authors:** Lorenzo Melchor, Laura Paula Saucedo-Cuevas, Iván Muñoz-Repeto, Socorro María Rodríguez-Pinilla, Emiliano Honrado, Alfredo Campoverde, Jose Palacios, Katherine L Nathanson, María José García, Javier Benítez

**Affiliations:** 1 Human Genetics Group, Human Cancer Genetics Program, Spanish National Cancer Research Center (CNIO), Madrid, E-28029, Spain; 2 Breast and Gynecological Group, Molecular Pathology Program, Spanish National Cancer Research Center (CNIO), Madrid, E-28029, Spain; 3 The Institute of Cancer SOLCA, Cuenca, Ecuador; 4 Department of Medicine, Medical Genetics, Abramson Cancer Center, University of Pennsylvania (UPENN), Philadelphia, PA-19104, USA; 5 Center for Biomedical Research on Rare Diseases (CIBERER), Calle de Alvaro de Bazan, 10 Bajo, 46010 Valencia, Spain; 6 Current address: The Breakthrough Breast Cancer Research Center, The Institute of Cancer Research (ICR), SW3 6JB, UK

## Abstract

**Introduction:**

Breast cancer subtypes exhibit different genomic aberration patterns with a tendency for high-level amplifications in distinct chromosomal regions. These genomic aberrations may drive carcinogenesis through the upregulation of proto-oncogenes. We have characterized DNA amplification at the human chromosomal region 13q34 in breast cancer.

**Methods:**

A set of 414 familial and sporadic breast cancer cases was studied for amplification at region 13q34 by fluorescence *in situ *hybridization (FISH) analysis on tissue microarrays. Defining the minimal common region of amplification in those cases with amplification at 13q34 was carried out using an array-based comparative genomic hybridization platform. We performed a quantitative real-time - polymerase chain reaction (qRT-PCR) gene expression analysis of 11 candidate genes located within the minimal common region of amplification. Protein expression levels of two of these genes (*TFDP1 *and *CUL4A*) were assessed by immunohistochemical assays on the same tissue microarrays used for FISH studies, and correlated with the expression of a panel of 33 antibodies previously analyzed.

**Results:**

We have found 13q34 amplification in 4.5% of breast cancer samples, but the frequency increased to 8.1% in *BRCA1*-associated tumors and to 20% in basal-like tumors. Tumors with 13q34 amplification were associated with high grade, estrogen receptor negativity, and expression of EGFR, CCNE, CK5, and P-Cadherin, among other basal cell markers. We have defined a 1.83 megabases minimal common region of genomic amplification and carried out mRNA expression analyses of candidate genes located therein, identifying *CUL4A *and *TFDP1 *as the most likely target genes. Moreover, we have confirmed that tumors with 13q34 amplification significantly overexpress CUL4A and TFDP1 proteins. Tumors overexpressing either CUL4A or TFDP1 were associated with tumor proliferation and cell cycle progression markers.

**Conclusions:**

We conclude that 13q34 amplification may be of relevance in tumor progression of basal-like breast cancers by inducing overexpression of *CUL4A *and *TFDP1*, which are both important in cell cycle regulation. Alternatively, as these genes were also overexpressed in non-basal-like tumor samples, they could play a wider role in cancer development by inducing tumor proliferation.

## Introduction

Breast cancer is a complex and heterogeneous disease, which is one of the most frequent causes of cancer deaths in developed countries. Most of breast cancer cases are sporadic; around 5% of breast cancer patients are considered as having hereditary breast cancer. These patients carry mutations in either *BRCA1 *[1] or *BRCA2 *[2] genes, but there also are familial breast cancer patients who do not carry mutations in *BRCA1*/*2 *and presumably have mutations in another unknown gene or gene(s) (termed non-*BRCA1/2 *or BRCAX patients). Over the last decade, many studies have shown that sporadic breast cancer can be grouped using molecular profiling into subtypes: basal-like, HER2-overexpressing, luminal A, and luminal B [3-5]. Researchers have identified molecular features that differentiate sporadic breast cancer from each group of familial breast cancers (*BRCA1*-, *BRCA2*-, or BRCAX-associated) [6-10]. However, our recent analyses have pointed out the striking similarities between sporadic and familial breast cancer in terms of the existence of breast cancer subtypes in both groups, as well as common patterns of genomic aberrations [11]. This finding may emphasize the interest in identifying molecular features that discriminate each of the sporadic and familial breast cancer subtypes, rather than comparing each group of familial breast cancer with sporadic tumors.

One common genomic aberration in breast cancer is high-level amplification. These aberrations may activate a profound increase in expression of genes within the amplification boundaries, crucial for the origin and progression of breast tumors. The most common regions of high-level amplification in breast cancer are 17q12 (targeting *ERBB2*), 11q13 (*CCND1*), 8q24 (*MYC*); 8p11-p12; 17q22-25; and 20q13 [12,13]. Interestingly, recent genomic analyses have shown that the chromosomal amplification sites tend to differ among the molecular breast cancer subtypes as for instance: 17q12 in HER2-overexpressing tumors, 20q13 in luminal-B tumors, 11q13 in both luminal A and B tumors, or 13q34 in basal-like tumors [5,11,14-16]. The definition of these chromosomal aberrations may elucidate genes crucial for the origin and progression of each breast cancer subtype. Taking this into account, we focused on a comprehensive characterization of 13q34 amplification (Amp13q34), a genomic aberration that we have recently found to be associated with basal-like breast cancers [11]. This amplification has been previously reported in squamous cell carcinomas [17], adrenocortical carcinomas [18], childhood medulloblastoma [19], hepatocellular carcinomas [20], and breast cancer [21], using conventional comparative-genomic hybridization (cCGH). Genes proposed to be the target genes of Amp13q34 include *CUL4A*, *LAMP1*, *TFDP1*, or *GAS6 *[20,22-26].

We have further characterized Amp13q34 in sporadic and familial breast cancer and defined its overall frequency, as well as its boundaries by fluorescence *in situ *hybridization (FISH) and array-based comparative genomic hybridization (aCGH) techniques. We have analyzed the gene- and protein-expression levels of candidate genes as well as their correlation with clinical and immunohistochemical (IHC) features. We propose that *CUL4A *and *TFDP1 *are likely the driver genes for this genomic amplification, leading higher tumor aggressiveness through deregulation of cell cycle.

## Materials and methods

### Patients and tumor samples

A total of 188 familial breast cancer patients belonging either to families with at least three women affected with breast and/or ovarian cancer, one of them diagnosed before 50 years of age, or to families with women affected with breast and/or ovarian cancer and at least one case of male breast cancer. All patients were screened for point mutations and large rearrangements in the *BRCA1/2 *genes using standard methods [27]. A series of 277 sporadic breast cancer samples came from the Spanish National Cancer Center (Spain) (172 samples), the Cancer Center of Solca (Ecuador) (86 cases), and the University of Pennsylvania (USA) (19). Written consent for tumor analyses and publication was obtained from the patient or their relative. This research study has been performed with the approval of an ethics committee.

### DNA isolation from formalin-fixed paraffin embedded tumor tissues

Genomic DNA isolation from formalin-fixed paraffin embedded (FFPE) tumors was carried out as previously described [28]. Briefly, two 30-μm sections were obtained from FFPE tumors, treated with xylene, incubated in Glycine Tris-EDTA and NaSCN, and finally digested with proteinase K and purified with phenol chloroform. All sections were previously examined and dissected with a scalpel to ensure at least 70% content of tumor cells.

### Array-based comparative genomic hybridization (aCGH)

Comparative genomic hybridization was done onto the 1 megabase (Mb) Bacterial Artificial Chromosome (BAC) clone array platform developed at the University of Pennsylvania. In few words, the platform is composed of 4134 BAC clones spaced at 1 Mb intervals, including a direct coverage of approximately 400 known cancer genes [29]. DNA probe labeling, aCGH protocol, and array data analysis have been described elsewhere [28]. Briefly, normalized aCGH data were analyzed using the Binary Segmentation algorithm implemented in the Insilico CGH software [30]. This algorithm depicts genomic segments showing estimative copy number values in log_2_ratio, which are calculated from the mean log_2_ratio of all the clones within that segment. As a result, it decreases both the dynamic range of the hybridization and the high noise usually present in hybridizations of DNA from FFPE tissues [31,32]. Therefore, we considered those segments with log_2_ratio ≥ 0.1 as gains, whereas those with log_2_ratio ≤ -0.1 were categorized as losses. Segments altered with DNA amplifications were considered when log_2_ratio ≥ 0.4. A collection of genomic imbalances previously confirmed in breast cancer cell lines and breast tumor samples allowed us to set these thresholds.

### Fluorescence *in situ *hybridization (FISH)

FISH analysis was carried out on the original group of six tumor samples with Amp13q34 found in the aCGH studies to verify their amplification levels. Then, we performed the FISH study on two familial and three sporadic breast cancer tissue microarrays (TMA) containing 156 and 258 breast tumor samples respectively, in order to quantify the Amp13q34 rate in a larger collection. From the familial breast cancer series, 44 samples were already included in our aCGH analysis [28]. These TMA have been described elsewhere [9,33,34]. Briefly, tumor areas of the breast tumor samples were carefully selected from hematoxilin and eosin-stained sections and delimited on the individual paraffin blocks. Then, two tissue cores from the selected tumor area of each specimen were obtained and included in the TMA.

The test FISH probe was composed of three BAC clones mapping to the 13q34 chromosomal region, labeled with dUTP-SpectrumOrange (Vysis, Inc. Downers Grove, IL, USA): RP11-391H12 (AL136221.38, mid-position 113.96 Mb), RP11-102K13 (AL160251.29, 114.09 Mb) and RP11-230F18 (AL442125.13, 114.21 Mb). In addition, three BAC clones from the 13q12.11 chromosomal region were used as copy number reference for chromosome 13, labeled with dUTP-SpectrumGreen (Vysis, Inc. Downers Grove, IL, USA): RP11-301J16 (AL137001.23, 19.63 Mb), RP11-408E5 (AL139327.18, 19.77 Mb), and RP11-385E5 (AL356259.11, 19.92 Mb). FISH analysis was done according to Vysis' instructions, with slight modifications. An average of 110 (50 to 200) well-defined nuclei was analyzed by scoring the number of single copy 13q34 and 13q12.11 signals. We considered DNA amplification when > 50% of tumor cells showed more than three times as many 13q34 signals as 13q12.11 copy signals.

### RNA isolation from formalin-fixed paraffin embedded tissues and cDNA synthesis

Total RNA was obtained from six breast tumors with Amp13q34 (three *BRCA1*-associated, one *BRCA2*-associated, one *BRCAX*-associated, and one sporadic breast tumor samples) and one breast cancer cell line that also carried the amplification (MDA-MB-157); as well as from 13 breast tumors that did not exhibit DNA copy number changes at 13q34. Importantly, 13q34 DNA copy number status for all tumoral specimens had been assessed by either aCGH or FISH analyses. All tumor samples were FFPE material, and RNA isolation was carried out as described before [35]. Briefly, a standard tissue sample deparaffinization was performed using xylene and alcohols. Then, samples were incubated in a digestion buffer (10 mM TrisHCl pH 8.0; 0.1 mM EDTA; 2% SDS and 500 μg/ml proteinase K). Nucleic acids were purified with standard phenol-chloroform-isoamyl alcohol procedures, followed by a precipitation with isopropanol, glycogen and sodium acetate. After DNase treatment (Ambion Inc., Austin, TX, USA) at 37°C for 30 minutes, RNA was finally processed for cDNA synthesis using M-MLV retrotranscriptase enzyme (Invitrogen, Carlsbad, CA, USA).

### Quantitative real time polymerase chain reaction (qRT-PCR) for mRNA expression

Assays were designed using the Roche Applied Science Universal Probe Library website [36] (Roche Diagnostics, Basel, Switzerland) for all target genes and endogenous control (see Additional file 1). qRT-PCR assays were set up in triplicates and performed using the ABI Prism 7900HT Sequence Detection System (Applied Biosystems, Foster City, CA, USA). Relative expression was determined using the free access software qBase [37] based on a modification of the classic delta-delta Ct method that allows for PCR efficiency correction. Appropriate positive and negative controls including non-retrotranscribed RNA for each sample were run for the experiment.

### Histology and immunohistochemistry

To associate genomic copy number aberration at 13q34 with changes in protein levels of the candidate genes, we carried out antibody specific staining on the same TMA used in the FISH analyses.

IHC assays were performed by the Envision method (Dako, Glostrup, Denmark) with a heat-induced antigen retrieval step. TMA sections were immersed in 10 mM boiling sodium citrate at pH 6.5 for two minutes in a pressure cooker. Between 150 and 200 cells per core were scored to determine the percentage of cells with positive nuclei or cytoplasm, depending upon the marker. We evaluated the protein expression level for the two candidate genes within Amp13q34 that were significantly overexpressed in the qRT-PCR analyses: *CUL4A *and *TFDP1*. As CUL4A expression in the cytoplasm was found in most of cases, we delineated four levels of staining: 0 (null staining), 1 (low staining), 2 (medium staining), and 3 (strong staining), which we grouped into negative (0), mid-level (1, 2) and positive (3) in subsequent analyses. TFDP1 is localized in the cell nucleus, so the median percentage of positive nuclei in all cases was used as the threshold to discriminate between negative (< 25%) or positive expression (≥ 25%). Tumor samples with more than 60% of stained cells (the median percentage of stained cells in only positive cases) were further categorized as highly expressed for the association with Amp13q34.

Other proteins also were studied to establish potential associations. We had previously evaluated nuclear staining for estrogen receptor (ER), progesterone receptor (PR), p53, Ki-67, cyclins D1, D3, E, and A; p16, p27, p21, CDK1, CDK2, CDK4, Skp2, retinoblastoma protein (Rb), E2F1, E2F6, MDM2, topoisomerase IIα, survivin, and CHEK2; cytoplasmic staining for BCL2, vimentin, cytokeratin 5/6 (CK5/6), cytokeratin 8 (CK8), and cyclin B1; and membrane staining for E-cadherin, P-cadherin, B-catenin and G-catenin [34]. HER-2 expression was evaluated according to the four-category (0 to 3+) DAKO system proposed for the evaluation of the HercepTest, and HER-2 expression of 3+ was the only value considered positive [34]. Antibodies, dilutions, suppliers, and thresholds used for analyses are listed in Additional file 2.

### Statistical analyses

In order to compare the mRNA expression levels between the different groups (tumors with or without Amp13q34), we applied the U-Mann Whitney test. We also determined associations between tumor groups with both clinical features and IHC markers using the chi square test, with the two-tailed Fisher's exact test correction when needed. The statistical software SPSS for Windows (SPSS, Inc., Chicago, IL, USA) was used to perform these statistical comparisons.

## Results

### Frequency and genomic definition of the 13q34 amplification

**Figure 1:**
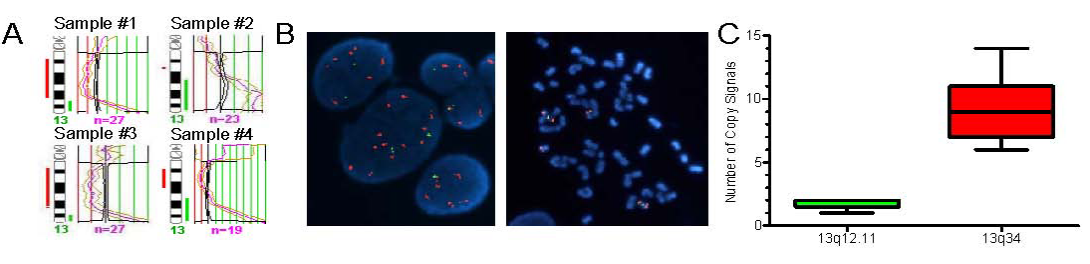
Genomic definition of the human 13q34 amplification I. **(a)** Chromosome 13 genomic profiles by conventional-comparative genomic hybridization (cCGH) of four breast cancer cases analyzed in a previous study by our group [21]. **(b)** FISH assays, detecting the 13q34 region (red probe) and the 13q12.11 region as a reference (green probe), show amplification at the 13q34 region in nuclei cell from a breast cancer sample (left, seven to eight red and one to two green signals) and in a metaphase of the MDA-MB-157 breast cancer cell line (right, six red and two green signals). **(c)** Boxplots displaying the range of copy signals for 13q12.11 (green box) and 13q34 (red box) in those cases with Amp13q34 found in our FISH screening.

We have previously reported that about 5% of both familial and sporadic breast cancers contain a high-level amplification at 13q34 (Amp13q34) using chromosomal- and array-based comparative genomic hybridization techniques (cCGH, and aCGH, respectively) (Table 1 and Figure 1a) [21,28]. Now, we have extended the study of the rate of Amp13q34 to a larger cohort of 414 familial and sporadic breast cancer samples using FISH technique. We have found that Amp13q34 is present in around 4.5% of breast cancer samples (Figure 1b-c), although the rate differs slightly among tumor classes, being higher in *BRCA1*- (8.1%) than in *BRCA2*- or non-*BRCA1*/*2*- (< 3.0%) associated cancers (Table 1). However, these differences are not statistically significant, maybe due to the low number of cases (data not shown). Noteworthy, the level of amplification in this tumor cohort never exceeded a 13q34:13q12.11 copy ratio of seven, being the median ratio of 4.5 as many 13q34 as 13q12.11 copy signals (Figure 1c). This shows that 13q34 region, albeit that it is affected with amplification, is not altered with the same magnitude as other classical high-level DNA amplification sites, such as 17q12 (*ERBB2*). As expected, no tumor sample analyzed by both cCGH/aCGH and FISH showed discrepancies in copy number values.

**Table 1:** DNA amplification rates at the region 13q34 in different analyses performed by our group

		DNA amplification rate % (*n*)				
		
Chromosomal region	Analysis	BRCA1	BRCA2	Non-BRCA1/2	Sporadic	Total
13q31-q34	cCGH*	**11.6 **(3/26)	**5.6 **(1/18)	**0 **(0/36)	-	**5.0 **(4/80)
13q34	aCGH**	**15.8 **(3/19)	**4.2 **(1/24)	**0 **(0/31)	**10.5 **(2/19)	**6.5 **(6/93)
13q34	FISH+	**9.5 **(2/21)	**0 **(0/14)	**4.8 **(3/66)	**4.2 **(8/192)	**4.4 **(13/293)
Total		**8.1 **(3/37)	**2.6 **(1/38)	**3.3 **(3/92)	**4.7 **(10/211)	**4.5 **(17/378)

The percentage of cases with Amp13q34 is shown.

*Melchor et al., 2005 [21]. Genomic amplification is considered when CGH ratio > 1.5 (see Figure 1a).

**Melchor et al., 2007 [28]. Amplification is regarded as genomic segments with log_2_ratio ≥ 0.4.

+Current study. Numbers correspond to cases in the tissue microarrays that were analyzable. We considered amplification when in tumor areas > 50% tumor cells showed > 3 times as many 13q34 signals as 13q12.11 copy signals.

**Figure 2:**
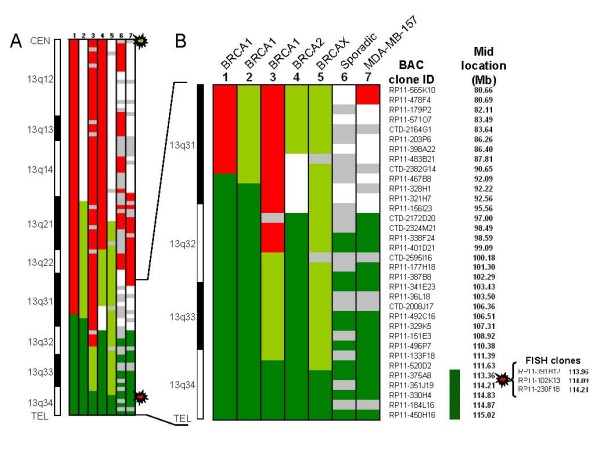
Genomic definition of the human 13q34 amplification II. **(a)** Genomic pattern of chromosome 13 by array-based comparative genomic hybridization (aCGH) in seven breast cancer samples (six familial breast tumors from the Spanish National Cancer Research Center, one sporadic breast tumor sample from the University of Pennsylvania, and MDA-MB-157 cell line) from centromere (top, CEN) to telomere (bottom, TEL). An idiogram showing chromosomal bands is depicted on the left. Each row represents a BAC array-clone sorted by their position in the UCSC Genome Browser (Human Feb 2009, assembly (hg 19)). Each clone is colored for each sample according to its level of genomic copies: loss (red), normal (white), gain (light green), amplification (dark green). Grey cells reflect clone data rejected after quality tests for signal intensity and replicate reproducibility. The green and red stars correspond to the location of the BAC probes used for FISH analyses. **(b)** aCGH genomic pattern of the 13q31-q34 chromosomal region. The BAC clone name and mid-position Mb is displayed. Colors mean the same as in A. The red star shows names and locations of the FISH clones used for the 13q34 probe. The striped dark green box represents the 1.83 Mb minimal common region of DNA amplification within the 13q34 site. A list of genes located within this minimal region is supplied in Table 2.

To define the minimal common region of amplification, we used data from a previous aCGH analysis [28]. A genomic representation of those cases with Amp13q34 (three *BRCA1*-, one *BRCA2*-, one BRCAX-, one sporadic-breast cancers and one breast cancer cell line, MDA-MB-157) is shown in Figure 2a. Chromosome 13 has two different genomic profiles based on the start position of the genomic gain: a) loss from the centromere to the 13q31 or 13q32 chromosomal band, with gain or amplification extending from 13q31 to the telomere (cases 1, 3, 4, 6, and 7); or b) copy neutral from the centromere to 13q21 and gain or amplification from 13q21 to the telomere (cases 2 and 5). A detailed analysis of the genomic values on each of the studied cases allowed us to narrow down the minimal common region of DNA amplification to 1.83 Mb, mapping entirely within the 13q34 chromosomal band. Two samples delimited the Amp13q34 region: tumor #3 showed the Amp13q34 starting at RP11-520D2 (spanning 3.55 Mb, and 29 genes), whereas tumor #5 finally defined the minimal 1.83 Mb region from RP11-375A8 to the telomere that contains 22 genes (Figure 2b) (Table 2). As tumor material was limited to carry out a whole characterization of all the genes therein located, we selected those related to tumorigenesis or cell transformation according to either previous studies or their hypothetical function. Our final list of selected genes included: *ARHGEF7*, *ATP11A*, *MCF2L*, *CUL4A*, *LAMP1*, GRTP1, *DCUN1D2*, *TFDP1*, *GAS6*, *RASA3*, and *CDC16 *(bold genes in Table 2). Although *ARHGEF7 *is not located within the boundaries of the 1.83 Mb region, it was selected to check a possible correlation for those genes located outside the minimal region, but inside the 3.55 Mb defined by the case #3. Noteworthy, the 13q34 FISH probe used in this study covered *CUL4A*, *LAMP1*, *GRTP1*, *DCUN1D2*, and *TFDP1 *(Figure 2 and Table 2).

**Table 2:** List of genes located in the minimal common region of amplification at 13q34

Gene Name*	Reference Sequence	Chromosome location (start pb - end pb)**	Name	Additional information
*ANKRD10*	NM 017664	111530888-111567416	ankyrin repeat domain 10	
***ARHGEF7***	**NM 145735.2**	**111767624-111947542**	**Rho guanine nucleotide exchange factor (GEF) 7**	**Rho GTPase**
*C13orf16*	NM 152324.1	111973015-111996593	chromosome 13 open reading frame 16	
*SOX1*	NM 005986.2	112721913-112726020	SRY (sex determining region Y)-box 1	Transcription factor involved in the regulation of embryonic development and in the determination of the cell fate
*C13orf28*	NM 145248.3	113030669-113089001	chromosome 13 open reading frame 28	
*TUBGCP3*	NM 006322.4	113139328-113242481	tubulin, gamma complex associated protein 3	
*C13orf35*	NM 207440.1	113301358-113338811	chromosome 13 open reading frame 35	
***ATP11A***	**NM 032189.3**	**113344643-113541480**	**ATPase, class VI, type 11A**	**Integral membrane ATPase**
***MCF2L***	**NM 001112732.1**	**113622757-113752862**	**MCF.2 cell line derived transforming sequence-like**	
*F7*	NM 000131.3	113760105-113774994	coagulation factor VII (serum prothrombin conversion accelerator)	Coagulation factor. Defects in this gene can cause coagulopathy
*F10*	NM 000504.3	113777113-113803841	coagulation factor X	Coagulation factor. Mutations of this gene result in factor X deficiency, a hemorrhagic condition of variable severity.
*PROZ*	NM 003891.1	113812968-113826694	protein Z, vitamin K-dependent plasma glycoprotein	
*PCID2*	NM 018386.2	113831925-113863029	PCI domain containing 2	
***CUL4A***	**NM 001008895.1**	**113863931-113919391**	**cullin 4A**	
***LAMP1***	**NM 005561.3**	**113951469-113977741**	**lysosomal-associated membrane protein 1**	**Membrane glycoprotein. It may also play a role in tumor cell metastasis**
***GRTP1***	**NM 024719.2**	**113978506-114018463**	**growth hormone regulated TBC protein 1**	
*ADPRHL1*	NM 138430.3	114076586-114107839	ADP-ribosylhydrolase like 1	Reversible posttranslational modification used to regulate protein function
***DCUN1D2***	**NM 001014283.1**	**114110134-114145023**	**DCN1, defective in cullin neddylation 1, domain containing 2 (S. cerevisiae)**	
*TMCO3*	NM 017905.4	114145308-114204542	transmembrane and coiled-coil domains 3	
***TFDP1***	**NM 007111.4**	**114239056-114295786**	**transcription factor Dp-1**	**Transcription factor that heterodimerizes with E2F proteins to enhance their DNA-binding activity and promote transcription from E2F target genes**.
*ATP4B*	NM 000705.2	114303123-114312501	ATPase, H+/K+ exchanging, beta polypeptide	Encodes the beta subunit of the gastric H+, K+-ATPase
*GRK1*	NM 002929.2	114321597-114438636	G protein-coupled receptor kinase 1	Ser/Thr protein kinase that phosphorylates rhodopsin and initiates its deactivation. Defects in GRK1 are known to cause Oguchi disease 2
*LOC100130386*	NR 028064.1	114451484-114454062	hypothetical protein LOC100130386	
***GAS6***	**NM 000820.2**	**114523524-114567046**	**growth arrest-specific 6**	**Gamma-carboxyglutamic acid (Gla)-containing protein thought to be involved in the stimulation of cell proliferation, and may play a role in thrombosis**
*FLJ44054*	NR 024609.1	114586610-114626485	hypothetical protein LOC643365	
***RASA3***	**NM 007368.2**	**114747195-114898095**	**RAS p21 protein activator 3**	**Member of the GAP1 family of GTPase-activating proteins. The gene product stimulates the GTPase activity of normal RAS p21 but not its oncogenic counterpart. Acting as a suppressor of RAS function, the protein enhances the weak intrinsic GTPase activity of RAS proteins resulting in the inactive GDP-bound form of RAS, thereby allowing control of cellular proliferation and differentiation**.
***CDC16***	**NM 003903.3**	**115000362-115038150**	**cell division cycle 16 homolog (S. cerevisiae)**	**Component protein of the APC complex, a cyclin degradation system that governs exit from mitosis**
*UPF3A*	NM 080687.1	115047078-115071281	UPF3 regulator of nonsense transcripts homolog A (yeast)	Component of a post-splicing multiprotein complex involved in both mRNA nuclear export and mRNA surveillance
*ZNF828*	NM_032436.2	115079965-115092802	zinc finger protein 828	

*Genes in bold were selected for candidate gene analyses by qRT-PCR.

**Gene locations are according to UCSC Genome Browser (Human Feb 2009, assembly (hg 19)).

### Analyses of the mRNA expression level for the candidate genes by qRT-PCR

**Figure 3:**
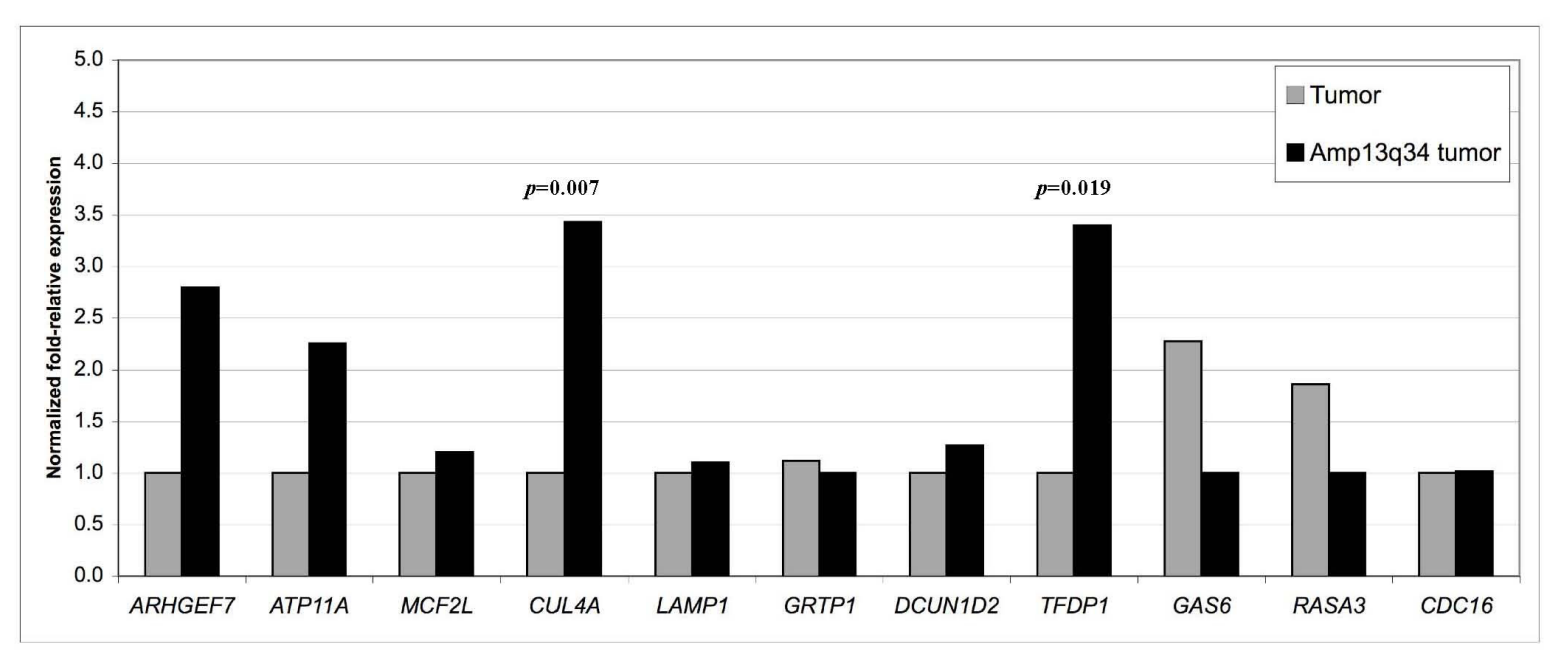
Gene expression analysis by qRT-PCR of the eleven candidate genes located in the minimal common regions of Amp13q34. Relative expression levels of the candidate genes in two sample cohorts: breast tumors with no genomic aberration at 13q34 (grey), and breast tumors with Amp13q34 (black). The expression of the candidate genes was evaluated by real-time quantitative RT-PCR and normalized with a housekeeping gene (*ACTB*). *P *values are shown for those genes with statistically significant differences when comparing the two groups in a U-Mann Whitney Test (*P *< 0.05).

Once the minimal amplification site had been delineated, qRT-PCR was carried out to determine the mRNA expression level of each of the 11 candidate genes, comparing two cohorts of FFPE samples: tumors with Amp13q34 (six tumor samples and MDA-MB-157), and tumors without any genomic aberration at 13q34 region (13 cases). *ARHGEF7 *and *ATP11A *showed a non-statistically significant trend to be overexpressed in Amp13q34 tumors (*P *= 0.242 and *P *= 0.191, respectively); whereas *GAS6 *and *RASA3 *had non-significant tendencies to be downregulated in tumors with Amp13q34 (*P *= 0.285 and *P *= 0.052, respectively) (Figure 3). Nevertheless, two genes had significant association between amplification and overexpression: *CUL4A *(*P *= 0.007) and *TFDP1 *(*P *= 0.019) (Figure 3). At this point, we focused on these two genes as candidate drivers of the 13q34 amplification.

### Correlation with protein expression levels and other immunohistochemical features

**Figure 4:**
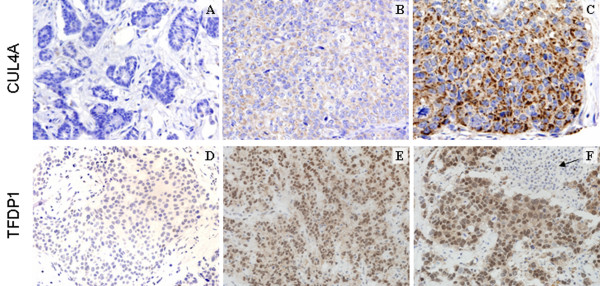
Immunohistochemical staining for CUL4A **(a-c)** and TFDP1 **(d-f) **in tumors with and without Amp13q34. (a) Null CUL4A staining in a non-amplifier tumor sample. (b) Moderate CUL4A staining in a non-amplifier tumor sample. (c) Strong CUL4A staining in a tumor with the Amp13q34. (d) Negative TFDP1 expression in a tumor without Amp13q34. (e) and (f) Strong TFDP1 expression in tumors with Amp13q34, the arrow in (f) points out the absence of expression in a lymphocyte infiltrate compared with the breast tumor cells.

We performed immunohistochemical analyses of the two candidate genes (*CUL4A *and *TFDP1*) on a set of 414 breast cancer samples on TMAs, previously analyzed by FISH (see above). Interestingly, the 75% of the tumors with Amp13q34 showed a strong staining for CUL4A, compared with 34.3% of the tumors without Amp13q34, but overexpressing CUL4A (*P *= 0.010) (Figure 4a-c and Table 3). A significant association was also found between Amp13q34 and TFDP1, since the 92.3% of the tumors with Amp13q34 (12/13) presented TFDP1 overexpression (*P *= 0.004), whereas the 51.9% of the tumors without Amp13q34 overexpressed TFDP1 (Figure 4d-f and Table 3). Furthermore, when considering a higher percentage (> 60%) of TFDP1 positive cells as the threshold, 76.9% of Amp13q34 tumors had TFDP1 overexpression as compared with 20.1% of the tumors without Amp13q34 (*P *= < 0.001) (Table 3). Interestingly, 60% of tumors with Amp13q34 co-overexpressed both CUL4A and TFDP1 (6 out of 10 tumors), although it was not statistically significant maybe due to the low number of samples (data not shown). Altogether, these findings demonstrated that Amp13q34 was significantly associated with a higher expression level of both CUL4A and TFDP1, but given that there are cases without the amplification showing protein overexpression, Amp13q34 was not the exclusive mechanism causing overexpression of these genes.

**Table 3:** Correlation of the presence/absence of Amp13q34 with the protein expression levels of CUL4A and TFDP1

	CUL4A expression					TFDP1 expression				
		
	CUL4A		CUL4A			TFDP1		TFDP1		
				
13q34	-ve	+ve	-ve	mid	+ve	-ve	+ve	-ve	+ve1	+ve2
No amp	155 (65.7)	81 (34.3)	4 (1.7)	151 (64.0)	81 (34.3)	115 (48.1)	124 (51.9)	115 (48.1)	76 (31.8)	48 (20.1)
Amp	3 (25.0)	9 (75.0)	0 (0)	3 (25.0)	9 (75.0)	1 (7.7)	12 (92.3)	1 (7.7)	2 (15.4)	10 (76.9)
*P*-values	*P *= 0.010		*P *= 0.017*			*P *= 0.004*		*P *= < 0.001*		

Each cell shows the number of samples and the percentage in the series between brackets.

CUL4A expression levels: a) -ve (negative) (null, low and medium staining) and +ve (positive) (strong staining); b) negative (null staining), mid (low and medium staining) and positive (strong staining).

TFDP1 expression levels: a) negative (< 25%) and positive (> 25%); b) negative (< 25% stained cells), +ve1 (positive) (between 25-60% stained cells), +ve2 (positive) (> 60% stained cells).

*P values from the Chi square of Pearson.

Amp = amplification.

In addition, we associated Amp13q34 with other immunohistochemical and clinical tumor features previously analyzed on a subset of the sample set (260 out of 414 samples) [33,34]. We observed that tumors with Amp13q34 were characterized by high histological grade; an absence of expression of ER, PR, CCND1, RB, p16, and CK8; and an overexpression of Ki-67, EGFR, P-Cadherin, G-catenin, CCNE, CCNB1, SKP2, survivin, vimentin, and CK5 (Table 4). As most of these markers are associated with basal-like phenotype of breast cancer, these associations may be due to the unique presence of the Amp13q34 in basal-like tumors as we previously reported [11]. CUL4A-overexpressing tumors had higher grade, absence of expression of PR and BCL2, and a higher percentage of cases expressing EGFR, P-Cadherin, CCNB1, SKP2, and CK5; when compared with tumors that did not overexpress CUL4A (Table 4). Last, TFDP1-overexpressing tumors were associated with overexpression of CCNE, CCNB1, and p16 (Table 4). However, no significant association was found between TFDP1 and CUL4A expression levels when studying the overall breast cancer sample set (data not shown). These associations support that the overexpression of CUL4A and TFDP1, even though it is correlated with Amp13q34, is not exclusive of basal-like tumors, but still relates to higher tumor aggressiveness.

**Table 4:** Protein marker associations between tumors with/without Amp13q34, CUL4A expression, or TFDP1 expression

Clinical & IHC feature	13q34 Amplification			CUL4A expression			TFDP1 expression		
			
	Absence	Presence	*P*	Negative	Positive	*P*	Negative	Positive	*P*
**Grade**									
1	42 (32.1)	0	0.002*	26 (40.6)	10 (19.2)	0.010*	12 (32.4)	27 (33.3)	NS*
2	40 (30.5)	0		19 (29.7)	13 (25.0)		11 (29.7)	22 (27.2)	
3	49 (37.4)	8 (100)		19 (29.7)	29 (55.8)		14 (37.8)	32 (39.5)	

**ER**									
Negative	56 (35.4)	8 (88.9)	**0.002**	30 (36.6)	29 (48.3)	NS	24 (46.2)	35 (38.0)	NS
Positive	102 (64.6)	1 (11.1)		52 (63.4)	31 (51.7)		28 (53.8)	57 (62.0)	

**PR**									
Negative	68 (47.2)	8 (88.9)	**0.018**	30 (43.5)	37 (62.7)	**0.034**	20 (48.8)	47 (52.3)	NS
Positive	76 (52.8)	1 (11.1)		39 (56.5)	22 (37.3)		21 (51.2)	42 (47.7)	

**BCL2**									
Negative	82 (56.9)	8 (88.9)	0.082	37 (53.6)	42 (71.2)	**0.047**	29 (70.7)	52 (58.4)	NS
Positive	62 (43.1)	1 (11.1)		32 (46.4)	17 (28.8)		12 (29.3)	37 (41.6)	

**Ki-67**									
0-5%	68 (47.2)	1 (11.1)	< 0.001*	35 (50.7)	22 (37.3)	NS*	22 (53.7)	35 (39.3)	NS*
6-25%	56 (38.9)	1 (11.1)		24 (34.8)	20 (33.9)		13 (31.7)	32 (36.0)	
> 25%	20 (13.9)	7 (77.8)		10 (14.5)	17 (28.8)		6 (14.6)	22 (24.7)	

**EGFR**									
Negative	181 (90.0)	4 (40.0)	**< 0.001**	122 (91.7)	57 (80.3)	**0.024**	94 (91.3)	89 (85.6)	NS
Positive	20 (10.0)	6 (60.0)		11 (8.3)	14 (19.7)		9 (8.7)	15 (14.4)	

**Cadherin P**									
Negative	128 (92.1)	3 (33.3)	**< 0.001**	63 (95.5)	46 (79.3)	**0.011**	37 (94.9)	74 (86.0)	NS
Positive	11 (7.9)	6 (66.6)		3 (4.5)	12 (20.7)		2 (5.1)	12 (14.0)	

**G-Catenin**									
Negative	106 (79.1)	4 (44.4)	**0.031**	59 (86.8)	43 (75.4)	NS	36 (90.0)	69 (78.4)	NS
Positive	28 (20.9)	5 (55.5)		9 (13.2)	14 (24.6)		4 (10.0)	19 (21.6)	

**Cyclin D1**									
Negative	67 (47.2)	8 (88.9)	**0.018**	32 (47.1)	27 (46.6)	NS	17 (42.5)	42 (47.7)	NS
Positive	75 (52.8)	1 (11.1)		36 (52.9)	31 (53.4)		23 (57.5)	46 (52.3)	

**Cyclin E**									
Negative	103 (72.5)	2 (22.2)	**0.004**	50 (73.5)	34 (58.6)	0.090	32 (80.0)	53 (60.2)	**0.043**
Positive	39 (27.5)	7 (77.8)		18 (26.5)	24 (41.4)		8 (20.0)	35 (39.8)	

**Cyclin B1**									
Negative	111 (80.4)	4 (44.4)	**0.024**	57 (86.4)	40 (70.2)	**0.045**	37 (92.5)	61 (72.6)	**0.010**
Positive	27 (19.6)	5 (55.6)		9 (13.6)	17 (29.8)		3 (7.5)	23 (27.4)	

**RB**									
Negative	33 (23.9)	6 (66.7)	**0.011**	12 (18.2)	14 (24.1)	NS	11 (27.5)	15 (17.4)	NS
Positive	105 (76.1)	3 (33.3)		54 (81.8)	44 (75.9)		29 (72.5)	71 (82.6)	

**E2F6**									
Negative	89 (64.5)	4 (44.4)	NS	39 (59.1)	38 (66.7)	NS	30 (75.0)	47 (56.0)	**0.049**
Positive	49 (35.5)	5 (55.6)		27 (40.9)	19 (33.3)		10 (25.0)	37 (44.0)	

**P16**									
Negative	53 (39.0)	7 (77.8)	**0.033**	24 (36.9)	30 (51.7)	NS	23 (59.0)	31 (36.0)	**0.020**
Positive	83 (61.0)	2 (22.2)		41 (63.1)	28 (48.3)		16 (41.0)	55 (64.0)	

**SKP2**									
Negative	66 (46.8)	1 (11.1)	**0.043**	40 (58.8)	22 (37.9)	**0.021**	24 (60.0)	40 (46.0)	NS
Positive	75 (53.2)	8 (88.9)		28 (41.2)	36 (62.1)		16 (40.0)	47 (54.0)	

**Survivin**									
Negative	97 (70.8)	1 (11.1)	**0.001**	49 (72.1)	35 (61.4)	NS	30 (75.0)	57 (64.8)	NS
Positive	40 (29.2)	8 (88.9)		19 (27.9)	22 (38.6)		10 (25.0)	31 (35.2)	

**CK5**									
Negative	133 (86.4)	3 (33.3)	**0.001**	74 (90.2)	43 (72.9)	**0.011**	42 (82.4)	77 (82.8)	NS
Positive	21 (13.6)	6 (66.6)		8 (9.8)	16 (27.1)		9 (17.6)	16 (17.2)	

**CK8**									
Negative	28 (19.9)	7 (77.8)	**0.001**	12 (17.6)	17 (29.3)	NS	7 (17.5)	24 (27.0)	NS
Positive	113 (80.1)	2 (22.2)		56 (82.4)	41 (70.7)		33 (82.5)	65 (73.0)	

**Vimentin**									
Negative	109 (79.0)	3 (33.3)	**0.006**	53 (77.9)	41 (70.7)	NS	32 (80.0)	63 (72.4)	NS
Positive	29 (21.0)	6 (66.6)		15 (22.1)	17 (29.3)		8 (20.0)	24 (27.6)	

ER = Estrogen Receptor; PR = Progesterone Receptor; CK = cytokeratin. RB = retinoblastoma gene protein.

Markers statistically significant in at least one of the tumor group comparisons are shown. *P *values ≤ 0.10 are depicted, in bold those statistically significant (*P *≤ 0.05). NS = non-significant. *P *values were calculated by Fisher's Exact Test, except histological grade and Ki-67 analyses that were done with Pearson's Chi square (*).

Other markers were also analyzed (such as HER2) but gave no significant associations.

## Discussion

In the present study, we have characterized the genomic amplification at the human 13q34 chromosomal region in familial and sporadic breast cancer cases. We have defined a minimal common region of amplification using FISH and aCGH techniques. Moreover, we have characterized the gene and protein expression levels of eleven candidate genes within the region using qRT-PCR and immunohistochemistry assays. The correlations that we have found suggest *TFDP1 *and *CUL4A *as likely drivers of this genomic aberration.

Amp13q34 previously has been reported in breast cancer [22,23] as well as in squamous cell carcinomas, adrenocortical carcinomas, childhood medulloblastoma, and hepatocellular carcinomas [17-20]. In our analyses, Amp13q34 is a low frequent genomic event in overall breast cancer (around 4.5%) but the rate increases when analyzing *BRCA1*-associated breast cancer (8.1%) (Table 1) or basal-like tumors (about 20%) [11]. Although this increase is not statistically significant (data not shown), it is in agreement with previous analyses that describe genomic gains/amplifications at 13q34 in ER-negative tumors, basal-like tumors, *BRCA1*-associated breast cancers, and medullary carcinomas [10,16,38,39]. Furthermore, we found that Amp13q34 is associated with a high histological grade, hormonal receptor negativity, and an overexpression of basal cytokeratins, cell cycle promoters (CCNE, CCNB1) and EGFR, among other markers (Table 4). These findings suggest that Amp13q34 is biologically important in the progression of basal-like breast cancers. Although a screening in a larger basal-like tumor cohort would be needed; once the prevalence of Amp13q34 is confirmed in this breast tumor phenotype, this genomic aberration could be used both as a clinical marker and as a therapeutic target for Amp13q34 tumors.

As far as we know, this is the first high-resolution genomic characterization of the amplification at the human 13q34 chromosomal site. We narrowed down the minimal common region of Amp13q34 to 1.83 Mb, which included 22 genes, of which 11 may be related to tumorigenesis: *ARHGEF7*, *ATP11A*, *MCF2L*, *CUL4A*, *LAMP1*, *GRTP1*, *DCUN1D2*, *TFDP1*, *GAS6*, *RASA3*, and *CDC16 *(Figure 2 and Table 2). In a recent study of mammary tumors arising in a p53-null mouse model, a genomic amplification at mouse chromosomal 8A1 region, syntenic to the human 13q34 region, was defined with a high-resolutive genomic BAC array. The authors reported two minimal clusters of amplification that also covered the aforementioned genes. In addition, they described amplification of *CUL4A *(25.7%), *LAMP1 *(13.5%), *TFDP1 *(31.1%), and *GAS6 *(13.5%) by DNA qRT-PCR on a set of 74 human breast carcinomas [22]. Noticeably, the amplification and overexpression of *CUL4A *was also reported in primary breast cancers a few years ago [23]. All these findings support a role for Amp13q34 in human breast cancer, likely through the increased expression of the genes located therein.

Based on the correlations observed in this study, *CUL4A *and *TFDP1 *are suggested to be the target genes for this genomic amplification. Both their mRNA (Figure 3) and proteins (Figure 4, and Table 3) are significantly overexpressed in breast tumors with Amp13q34 when compared with breast tumors without 13q34 genomic alterations. Therefore, our data support the importance of these genes as targets for the amplification, consistent with previous findings not only in breast cancer [22], but also in hepatocellular carcinomas [20].

*CUL4A *is a member of the cullin protein family and composes the multifunctional ubiquitin-protein ligase E3 complex [40]. Our results suggest that *CUL4A *may play a role in breast cancer progression, based on its amplification and significant overexpression, previously reported in breast malignancies [22,23], as well as in other carcinomas [20]. It has been shown that CUL4A could contribute to tumor malignancy mediating the activity of tumor suppressors and thus, altering cell cycle checkpoints [41-44]. Moreover, a recent study has reported that breast cancer patients with strong expression of CUL4 had a significantly shorter overall and disease-free survival [25]. In our analysis, CUL4A overexpressing tumors were characterized by a high histological grade, an absence of PR and BCL2 expression, and a high expression of cell cycle promoters (cyclin B and SKP2), cell signaling components (EGFR), basal cytokeratins (CK5), and cell adhesion proteins (P-cadherin) (Table 4), suggesting high cell proliferation and tumor aggressiveness. As some of these features are basal-cell markers (such as EGFR, CK5, P-Cadherin), CUL4A may play an important role in the biology of basal-like breast cancers. Furthermore, our results suggest the role for *CUL4A *as part of the development of breast tumors in general, mainly through deregulation of cell cycle checkpoints.

TFDP1 is a heterodimerization partner for members of the E2F family of transcription factors. E2F1/TFDP1 forms a complex involved in cell cycle progression by the regulation of expression of cell cycle promoters (cyclin A, cyclin E, CDK2) [45]. In our study, breast tumors overexpressing TFDP1 had high expression of p16, cyclin E and cyclin B1 (Table 4), as described in hepatocellular carcinoma [20]. These associations point out a deregulation of the cell cycle in these tumors. However, overexpression of TFDP1 did not correlate with altered expression levels either of its protein partner, E2F1, or one of the E2F1/TFDP1 regulators, RB, in our tumor cohort (data not shown). The lack of correlation between TFDP1 and E2F1 expression suggests that TFDP1 may have a function in addition to gene transcription regulation and cell cycle progression in breast cancer. This discordance between E2F1 and TFDP1 protein levels was also described in non-Hodgkin lymphomas [46] and in hepatocellular carcinomas, where only TFDP1 overexpression was associated with a larger tumor size [26]. Additionally, Abba and colleagues analyzed publicly available human breast cancer mRNA expression datasets and found significant associations of TFDP1 overexpression with shorter overall survival, relapse-free survival, and metastasis-free interval [22]. Moreover, TFDP1 overexpression has been related to progression of hepatocellular carcinomas [26], and together with activated HA-RAS, causes oncogenesis in rat embryo fibroblasts [47]. Thus, *TFDP1 *is not only a strong candidate to drive Amp13q34, but also a gene involved in different components of tumorigenesis, causing higher tumor aggressiveness and a poor patient prognosis. Further functional analyses to elucidate its potential role in breast cancer oncogenesis need to be performed.

The other candidate genes that we studied (*ARHGEF7*, *ATP11A*, *MCF2L*, *LAMP1*, *GRTP1*, *DCUN1D2*, *GAS6, RASA3 *and *CDC16*) did not demonstrate overexpression in Amp13q34 tumors, although non-significant trends were found for overexpression of *ARHGEF7 *and *ATP11A*, and downregulation of *GAS6 *and *RASA3 *(Figure 3). However, the lack of significant correlations for these genes between copy number and expression in our dataset should not rule out their possible role in breast cancer. For example, a recent article associated expression of *GAS6 *with indicators of good prognosis such as progesterone receptor positivity, small tumor size, low grade, and young patient age [24]. These tumor features do not correspond to the ones found in Amp13q34 tumors, which are mainly ER and PR negative (Table 4); thus, *GAS6 *may not be the target gene in the Amp13q34, but may still play a role in other breast cancers.

## Conclusions

In summary, we have characterized a 1.83 Mb minimal common region of amplification at 13q34 that may play a crucial role in the development of basal-like breast cancers. Analyses of larger series of this tumor subtype will allow us to confirm the specificity of this aberration and, importantly, its possible use as a clinical marker of basal-like tumors. This genomic aberration could facilitate the tumor progression through the overexpression of driver/target genes such as *TFDP1 *and *CUL4A*, which are overexpressed not only in Amp13q34 tumors, but also in other breast cancer samples characterized by a high cell proliferation and aggressiveness. Therefore, further functional analyses should be performed to demonstrate their potential oncogenic roles, so that the development of pharmaceutical suppressors of CUL4A or TFDP1 activity could provide a therapeutic target for tumors overexpressing these proteins.

## Abbreviations

aCGH: array-based comparative genomic hybridization; Amp13q34: amplification at human chromosomal 13q34 region; BAC: Bacterial Artificial Chromosome; cCGH: conventional or chromosomal comparative genomic hybridization; CK: cytokeratin; ER: estrogen receptor protein; FFPE tumors: formalin-fixed paraffin embedded tumors; FISH: fluorescence *in situ *hybridization; IHC: immunohistochemical; Mb: megabase(s); PR: progesterone receptor protein; qRT-PCR: quantitative real time polymerase chain reaction; RB: retinoblastoma gene or protein; TMA: tissue microarray.

## Competing interests

The authors declare that they have no competing interests.

## Authors' contributions

LM carried out the genomic and qRT-PCR analyses, performed the statistical analyses and drafted the manuscript. LPSC carried out the qRT-PCR assays. Pathologists IMR, SMRP, EH and JP analyzed the immunoassays. AC and JP provided tumor sample sets for the study. KLN provided the array-CGH platform and helped to draft the manuscript. MJG participated in the design of the study. JB conceived of the study, participated in its design and coordination, and helped to draft the manuscript. All authors read and approved the final manuscript.

## Supplementary Material

Additional file 1 Word document containing a table showing genes, primers and Universal Probe Library from Roche^© ^used for qRT-PCR analyses in the present study. Click here for file

Additional file 2 Word file containing a table listing the antibodies used in the present immunohistochemical analysis and thresholds established to consider a tumor as positive. Click here for file
