# Supplementary material for: Comprehensive characterization of the DNA amplification at 13q34 in human breast cancer reveals TFDP1 and CUL4A as likely candidate target genes
Source: Breast Cancer Res. 2009 Dec 8;11(6):R86. doi: 10.1186/bcr2456 (PMC2815550; doi:10.1186/bcr2456)
Supplement: Additional file 1 — Word document containing a table showing genes, primers and Universal Probe Library from Roche© used for qRT-PCR analyses in the present study. [file bcr2456-S1.doc]

**Additional Data File 1**. Genes, primers and Universal Probe Library from Roche© used for qRT-PCR analyses.

| **Gene Name** | **Target or Endogenous Gene (T/E)** | **Primers** | | **Universal Probe #** |
| --- | --- | --- | --- | --- |
| *ARHGEF7* | T | Fw | caacatccgcgagttcct | #19 |
|  |  | Rv | ctgggaaggaggaaacagg |  |
| *ATP11A* | T | Fw | tggtgctacagctgttgagg | #2 |
|  |  | Rv | agggcctcgatggtgtct |  |
| *MCF2L* | T | Fw | gctggagcagtcacagagc | #11 |
|  |  | Rv | ggcttagggggtctgttttc |  |
| *CUL4A* | T | Fw | ggaaagcacagtggtcgaa | #59 |
|  |  | Rv | gggacacctggaattccttc |  |
| *LAMP1* | T | Fw | acctggtgactctggagctg | #15 |
|  |  | Rv | accggctagaacttgcattc |  |
| *GRTP1* | T | Fw | agctacctggtcacgctcac | #66 |
|  |  | Rv | cggacatagcgcttcactg |  |
| *DCUN1D2* | T | Fw | ggaacgctgtggacaagaa | #78 |
|  |  | Rv | tttgttttcatcttgtggatcttt |  |
| *TFDP1* | T | Fw | cacgtctaacggcacaagg | #53 |
|  |  | Rv | ctgagacccattggagcttg |  |
| *GAS6* | T | Fw | atggcatgtggcagacaat | #17 |
|  |  | Rv | ccctgttgaccttgatgacc |  |
| *RASA3* | T | Fw | ggcagtccagctcctacg | #19 |
|  |  | Rv | ggctcttgctaccattgtcc |  |
| *CDC16* | T | Fw | cagcagcacagctgatgaa | #41 |
|  |  | Rv | agggtcttccggtgcaat |  |
| *B-ACT* | E | Fw | ccaaccgcgagaagatga | #64 |
|  |  | Rv | ccagaggcgtacagggatag |  |
