# Supplementary material for: Comprehensive characterization of the DNA amplification at 13q34 in human breast cancer reveals TFDP1 and CUL4A as likely candidate target genes
Source: Breast Cancer Res. 2009 Dec 8;11(6):R86. doi: 10.1186/bcr2456 (PMC2815550; doi:10.1186/bcr2456)
Supplement: Additional file 2 — Word file containing a table listing the antibodies used in the present immunohistochemical analysis and thresholds established to consider a tumor as positive. [file bcr2456-S2.doc]

**Additional Data File 2.** Antibodies used in the present immunohistochemical study and thresholds to consider a tumor as positive

| **Antibody** | **Clone** | **Dilution** | **Supplier** | **Threshold (%)** |
| --- | --- | --- | --- | --- |
| ER | 1D5 | 1:30 | Novocastra | 10 |
| PR | 1A6 | 1:30 | Novocastra | 10 |
| BCL2 | 124 | 1:80 | DAKO | 70 |
| Ki-67 | MIB1 | 1:30 | DAKO | 0-5/6-25/>25 |
| EGFR | EGFR.113 | 1:10 | Novocastra | * |
| HER-2 | Herceptest | Prediluted | DAKO | 3+ |
| Cyclin D1 | DCS-6 | 1:100 | DAKO | 30 |
| Cyclin D3 | DCS-22 | 1:10 | Novocastra | * |
| Cyclin E | 13A3 | 1:10 | Novocastra | * |
| Cyclin A | 6E6 | 1:100 | Novocastra | * |
| Cyclin B1 | 7A9 | 1:25 | Novocastra | * |
| P53 | DO-7 | 1:50 | Novocastra | 25 |
| p21 | EA10 | 1:50 | Oncogene | * |
| p16 | Poly mouse | 1:50 | Santa Cruz | 50 |
| p27 | 57 | 1:1000 | Transduction Lab | 50 |
| Cdk1 | 1 | 1:1500 | Transduction Lab | * |
| Cdk2 | 8D4 | 1:500 | NeoMarkers | * |
| Cdk4 | 35.1 | 1:10 | Chemicon | * |
| Skp2 | 1G12E9 | 1:10 | ZYMED | * |
| Rb | G3-245 | 1:250 | BD PharMingen | * |
| E2F1 | KH20-KH95 | 1:200 | Upstate | 0-46/46-55/>55 |
| E2F6 | Poly goat | 1:50 | Santa Cruz | * |
| CHEK2 | DCS-270 | 1:25 | Novocastra | 60 |
| Topoisomerase IIα | Ki-S1 | 1:400 | DAKO | * |
| MDM2 | IF2 | 1:10 | Oncogen | * |
| CK 5/6 | D5/16 B4 | 1:25 | DAKO | * |
| CK 8 | 35BH11 | 1:10 | DAKO | 80 |
| Vimentin | V9D | 1:500 | DAKO | * |
| Survivin | Poly rabbit | 1:1000 | RD Systems | * |
| E-Cadherin | 4A2C7 | 1:200 | Zymed | * |
| P-Cadherin | 56 | 1:200 | Transduction Lab | 10 |
| B-Catenin | 14 | 1:1000 | Transduction Lab | * |
| γ-Catenin | 15 | 1:1000 | Transduction Lab | * |
| Cul4a | 10693-1-AP | 1:25 | Proteintech Group | 0/1/2/3 ** |
| Tfdp1 | 1DP06 | 1:100 | NeoMarkers | 25 |

* Any positive cell.

** Intensity of staining level, as cell staining is present in most of cases.
